# 20 Jahre nach 9/11 – Wie zukunftsfähig ist die Außenpolitik der Europäischen Union?

**DOI:** 10.1007/s41358-021-00293-0

**Published:** 2021-11-02

**Authors:** Carolin Rüger

**Affiliations:** Professur für Europaforschung und Internationale Beziehungen, Institut für Politikwissenschaft und Soziologie, Julius-Maximilians-Universität Würzburg, Wittelsbacherplatz 1, 97074 Würzburg, Deutschland

## Weckruf vom Hindukusch

Die jüngsten Ereignisse in Afghanistan seien ein „Weckruf“ für die Europäische Union (EU), so formulierte es Josep Borrell, Hoher Vertreter der Union für Außen- und Sicherheitspolitik in der *New York Times* am 1. September 2021, fast 20 Jahre nach den verheerenden Terroranschlägen in den USA. 9/11 stellt als „wahrer Beginn des 21. Jahrhunderts“ (Garton Ash 2010, S. 220) eine historische Zäsur dar und bleibt bis heute, so der Präsident des Europäischen Parlaments David Sassoli, eine „offene Wunde in unser aller Herzen“. Der auf die Anschläge folgende US-geführte ‚Krieg gegen den Terror‘ fand mit dem Abzug aus Afghanistan ein erschütterndes Ende. Die dramatischen Bilder der Evakuierungen nach der Machtübernahme der Taliban ab Mitte August 2021 zeichnen auch ein unrühmliches Bild der EU. Eine EU-weite Koordination der Rettung von Ortskräften und Unionsbürgern und -bürgerinnen gab es nicht. Inmitten des „schluchzenden Westens“ (Bonschab und Kappel 2021) war Europa maximal ein hilfloser Zaungast, der sicherheitspolitisch nichts zu bieten hat und trotz jahrelanger Debatten über strategische Autonomie nicht in der Lage ist, einen Flughafen ohne Hilfe der USA zu sichern. Besonders fatal erscheint die europäische Hilflosigkeit, da die EU an der Stabilität in Afghanistan ein essenzielles Interesse hat – nicht zuletzt wegen potenzieller Migrationsbewegungen. Die EU-Mitgliedstaaten waren geeint in einer Mischung aus Angst, Schock und Entsetzen darüber, dass ihr zwei Jahrzehnte lang geleistetes, finanzielles sowie von Militär- und Zivilkräften im Einsatz getragenes Engagement im Taliban-Terror endete. Dass auch andere Akteure, allen voran die USA, kein besseres Bild abgaben, ist kaum ein Trost. „Gemeinsam rein, gemeinsam raus, gemeinsam gescheitert“: Das „Afghanistan-Fiasko“ (Riegert 2021) steht sinnbildlich für das Versagen des Westens insgesamt, aber auch im Speziellen der EU. Ist damit alles über die Zukunftsfähigkeit der EU-Außenpolitik gesagt? Oder kann der Weckruf vom Hindukusch als Katalysator für die Rolle der EU in der Weltpolitik wirken? Dieser Beitrag soll das globale Handeln der EU auf seine Zukunftsfähigkeit im 21. Jahrhundert hin abklopfen. Dabei geht es nicht darum, eine Bilanz der EU-Afghanistanpolitik vorzulegen oder die generelle Rolle der EU im Kampf gegen den internationalen Terrorismus zu bewerten. Die Kurzanalyse verfolgt stattdessen das Ziel, das ‚große Ganze‘ der EU-Außenpolitik schlaglichtartig zu beleuchten. Wie ist es um deren aktuelle Verfasstheit in der post-Afghanistan-Ära bestellt, und wie ist die EU für die globalen Herausforderungen in einer Welt im Umbruch gerüstet?

## Konzeptualisierung der EU-Außenpolitik

Zunächst gilt es, die Außenpolitik der EU knapp zu konzeptualisieren. Sie wird oft gleichgesetzt mit der Gemeinsamen Außen- und Sicherheitspolitik (GASP), also der seit dem Vertrag von Maastricht bestehenden diplomatischen und politischen Zusammenarbeit der Mitgliedstaaten. Ihr sicherheitspolitischer Arm, die Gemeinsame Sicherheits- und Verteidigungspolitik (GSVP), resultierte Ende der 1990er-Jahre aus der Ohnmacht auf dem Balkan, wo die EU, so der ehemalige Kommissionspräsident Jacques Delors, „a child confronted with an adult crisis“ war. Oft erwecken GASP und GSVP den Eindruck, das „G“ im Namen stehe eher für „gespalten“ als für „gemeinsam“. Die schwarze Liste der Fehlschläge ist lang (Rüger 2016) und reicht vom Sündenfall in der ‚Causa Irak‘ 2003 über die Kakophonie in Libyen 2011 bis hin zu den oft divergenten Haltungen der Mitgliedstaaten im Nahen Osten sowie gegenüber China oder Russland. GASP und GSVP sind zweifelsohne zentrale Bausteine der EU-Außenpolitik. Eine analytische Fokussierung nur auf diese Politikbereiche würde die globale Rolle der EU jedoch unzulässig verkürzen. Die Außenpolitik der EU ist vielmehr als mehrdimensionales Mosaik zu betrachten (Müller-Brandeck-Bocquet und Rüger 2015), das neben der intergouvernementalen Dimension der GASP und GSVP weitere essenzielle Bausteine umfasst: Hierzu zählt zum einen die Gemeinschaftsdimension, bestehend aus Handelspolitik, Entwicklungszusammenarbeit und Humanitärer Hilfe. Diese supranational geprägte Dimension unterscheidet sich durch die Gemeinschaftsmethode fundamental von der weitgehend intergouvernementalen Governance der GASP und der GSVP. Die so genannten restriktiven Maßnahmen der EU stellen als eigene Dimension eine Mischform aus intergouvernementaler und supranationaler Sphäre dar. Diese werden einstimmig als Instrument der GASP verhängt, aber oft mit Mitteln aus der Gemeinschaftsdimension, insbesondere über handelspolitische Maßnahmen, implementiert. Einen weiteren Baustein der EU-Außenpolitik bilden die Erweiterungspolitik und die Europäische Nachbarschaftspolitik (sui-generis-Dimension). Komplettiert wird das Mosaik schließlich von der externen Dimension interner Politikbereiche. Hierzu zählen die Klimapolitik ebenso wie die externen Implikationen des Raums der Freiheit, der Sicherheit und des Rechts wie beispielsweise die Migrationspolitik oder die Terrorismusbekämpfung. Auch die mannigfaltigen Aktivitäten der EU zur globalen Regulierung der Digitalisierung sind Teil dieser oft unterschätzten Dimension.

Mit dieser multidimensionalen Perspektive ermöglicht es das Mosaik-Konzept der EU-Außenpolitik, deren komplexe und über Jahrzehnte gewachsene Vielgestaltigkeit in ihrer Gesamtheit zu erfassen und damit ein realitätsgerechtes und umfassendes Bild der globalen Rolle der EU heute und in der Zukunft zu gewinnen.

## Die Zukunft der EU-Außenpolitik im globalen Wettbewerb: eine SWOT-Kurzanalyse

Ein fundamentales Dilemma der internationalen Beziehungen des 21. Jahrhunderts besteht darin, dass die globalen Herausforderungen nur durch Kooperation zu bewältigen sind, die Welt und ihre Akteure jedoch zunehmend in den „Aggregatzustand“ des Wettbewerbs verfallen (Munich Security Conference 2021). Zur Positionsbestimmung eines Akteurs ist aus der Betriebswirtschaftslehre das Konzept der SWOT-Analyse bekannt. Dieses verbal-argumentative Bewertungsinstrument (Wollny und Paul 2015, S. 190) berücksichtigt sowohl die internen Stärken (***S****trengths*) und Schwächen (***W****eaknesses*) eines Akteurs als auch die externen Chancen (***O****pportunities*) und Risiken (***T****hreats*), die sich aus dem jeweiligen Wettbewerbs- und Handlungsumfeld ergeben. Es kann an dieser Stelle keine allumfassende Analyse vorgenommen werden. Eine 10-Punkte-SWOT-Kurzanalyse soll jedoch je drei zentrale interne Stärken und Schwächen des globalen Akteurs EU sowie je zwei externe Möglichkeiten und Bedrohungen der EU-Außenpolitik konturieren. Leitschnur und Bewertungsmaßstab ist dabei das Kriterium der Zukunftsfähigkeit der EU im globalen Wettbewerb. Diese Zukunftsfähigkeit wird im Folgenden verstanden als Weltpolitikfähigkeit im Sinne des ehemaligen Kommissionspräsidenten Jean-Claude Juncker: „die Fähigkeit, die Geschicke der Welt als Union mitzugestalten“.

### Stärken

Wie oben bereits angesprochen, gerät beim Fokus auf GASP- und GSVP-Zwistigkeiten leicht aus dem Blick, welches Gewicht die EU heute schon in die weltpolitische Waagschale werfen kann. Die Redewendung, die EU sei ein wirtschaftlicher Riese, ein politischer Zwerg und ein militärischer Wurm ist altbekannt und vor allem mit Blick auf die geoökonomische Position der Union weiterhin gültig: Die EU ist auch nach den wirtschaftlichen Verwerfungen der jüngeren Krisen und nach dem Brexit eine Weltwirtschaftsmacht auf Augenhöhe mit den USA und China, der Euro ist neben dem Dollar globale Leitwährung, die EU und ihre Mitgliedstaaten leisten das Gros der weltweiten Entwicklungshilfezahlungen und können über den Hebel der Konditionalisierung transformative Macht (Grabbe 2006; Börzel und Risse 2009) ausüben. „Leise Supermacht“ (Rifkin 2005), „sanfte Supermacht“ (Fichtner 2021), „invisible superpower“ (Moravcsik 2017) – diese Attribute deuten an, dass die EU *soft power* ausübt, deren Wirkkraft gemeinhin unterbewertet wird. Der von Anu Bradford (2020) eindrucksvoll analysierte und an einer Fülle von Beispielen ausbuchstabierte „Brüssel-Effekt“ belegt, dass die EU allein durch die Kraft ihres Binnenmarktes, durch die hohe Kaufkraft der europäischen Verbraucher und Verbraucherinnen globale Standards setzt. Die EU ist somit – oft unbemerkt – ein „Gigant, der das Leben auf Erden entscheidend mitgestaltet“ (Fichtner 2021), und schafft als *soft power* weltweit harte regulatorische Fakten. Die politische und militärische Macht kann (noch) nicht Schritt halten mit der ökonomischen, doch auch hier ist ein Trend nach oben erkennbar.^1^

Neben der realiter vorhandenen globalen Wirtschafts- und Gestaltungsmacht, verfügt die EU – dies ist der zweite Punkt auf der Habenseite – über einen reich gefüllten Werkzeugkasten. Damit sind zum einen die oben aufgefächerten unterschiedlichen Dimensionen der außenpolitischen Handlungsbereiche angesprochen, zum anderen der zivil-militärische Ansatz der EU beim Krisenmanagement. Die EU verfolgt als „Zivilmacht mit Zähnen“ (Frank-Walter Steinmeier), einen dualen Ansatz, der zu ihrem „Markenzeichen“ (Joschka Fischer) geworden ist und sie gewissermaßen als *unique selling point* von anderen Akteuren wie der rein militärischen NATO grundlegend unterscheidet. Wenn US-Präsident Joe Biden einen Tag nach dem endgültigen Abzug der USA als Lehre aus Afghanistan festhält, dass Menschenrechte „not through endless military deployments, but through diplomacy, economic tools, and rallying the rest of the world for support“ zur Geltung gebracht werden müssen, klingt das sehr europäisch. Der von Biden gezeichnete Abschied von einem *boots-on-the-ground*-Ansatz zugunsten von mehr regionaler Diplomatie, Einflussnahme durch Hilfsleistungen und Prävention deckt sich stark mit europäischen Konzepten. Für die hybriden Sicherheitsbedrohungen unserer Zeit ist die EU-Außenpolitik mit ihrem einzigartigen Instrumentenmix zumindest potenziell gut gerüstet. Über die Bereitschaft, diese Instrumente auch einzusetzen, wird im weiteren Verlauf noch zu sprechen sein.

Drittens ist die EU ein vortreffliches Beispiel dafür, dass Kooperation unter widrigsten Umständen möglich ist. Den Friedensnobelpreis hat sie 2012 für ihre Leistung erhalten, aus einem Kontinent des Krieges einen Kontinent des Friedens gemacht zu haben. Artikel 21 EUV ist kodifizierter Anspruch der Innen-Außen-Analogie (Kittel et al. 1995, S. 68; Peters und Wagner 2005) der EU: „Die Union lässt sich bei ihrem Handeln auf internationaler Ebene von den Grundsätzen leiten, die für ihre eigene Entstehung, Entwicklung und Erweiterung maßgebend waren und denen sie auch weltweit zu stärkerer Geltung verhelfen will“. Demokratie, Rechtsstaatlichkeit, Menschenrechte und Grundfreiheiten, Gleichheit, Solidarität zählen ebenso zu diesen internalisierten Prinzipien wie der Multilateralismus, der – so formulierte es der ehemalige Kommissionspräsident José Manuel Barroso – Teil der „DNA der Union“ ist. Mit ihrem *en gros* sehr erfolgreichen multilateralen Modell zur Befriedung jahrhundertelanger Feindschaften durch Institutionalisierung, Verregelung und Verrechtlichung der Beziehungen hat die EU Modellcharakter für die Gestaltung und Zivilisierung der internationalen Beziehungen insgesamt.

### Schwächen

Den drei hier aufgeführten Stärken stehen gewichtige Schwächen des globalen Akteurs EU entgegen: An erster Stelle ist der „politische Wille“ zu nennen, dessen Fehlen in nahezu jeder Abhandlung zur EU-Außenpolitik konstatiert wird. Die EU-Außenpolitik ist von dem kontinuierlichen Zwiespalt gekennzeichnet, dass sich in den Mitgliedstaaten zwar inzwischen ein „Problemlösungsinstinkt“ durchgesetzt hat, wonach die europäische Ebene als optimal für die Bearbeitung gemeinsamer, grenzüberschreitender Herausforderungen perzipiert wird; gleichzeitig bleiben die mitgliedstaatlichen Akteure einem „Souveränitätsreflex“ verhaftet, um das Risiko der Aushöhlung nationaler Souveränität zu limitieren (Hofmann und Wessels 2008, S. 4). Gerade in den souveränitätssensiblen Bereichen der EU-Außenpolitik führt der fehlende politische Wille in der Konsequenz dazu, dass die EU-Staaten zwar insgesamt hohe Verteidigungsausgaben tätigen, ihre Kräfte aber noch zu wenig bündeln, dass *Battlegroups* bereitstehen, aber nie zum Einsatz kamen oder dass Europa bei wichtigen Fähigkeiten wie etwa dem strategischen Lufttransport oder der Aufklärung nach wie vor eklatante Lücken aufweist. Fehlender politischer Wille ist jedoch kein exklusives Problem der GSVP. Die in Sonntagsreden oft beschworene europäische Gemeinsamkeit ist bei den Montagstreffen der Außenminister und Außenministerinnen, wenn es beispielsweise um die schnelle Verabschiedung von Sanktionen geht, oft vergessen.

Ein zweites, gravierendes Bündel von Defiziten ist im institutionellen Bereich zu verorten. Die Governance der EU-Außenpolitik ist durch die Aufteilung auf die verschiedenen Dimensionen mit jeweils unterschiedlicher Genese der Politikbereiche und unterschiedlichen Pfadabhängigkeiten stark fragmentiert. Gleiches gilt nach wie vor für die Außenvertretung, wie der diplomatische Eklat in Ankara (‚Sofagate‘) erkennen ließ. Als größte institutionelle Achillesferse muss jedoch das Festhalten am Einstimmigkeitsprinzip im harten Kern der EU-Außenpolitik betrachtet werden. Die nationale Vetomöglichkeit in zentralen Fragen der Außenpolitik behindert nicht nur effiziente Beschlussfassungen, sondern macht die EU auch vulnerabel gegenüber einer *divide-et-impera*-Strategie externer Akteure, zu beobachten etwa 2017, als China Griechenland finanzielle Hilfen in Aussicht stellte und sich im Gegenzug das griechische Veto gegen eine Verurteilung im Menschenrechtsrat der Vereinten Nationen sicherte. Von souveränitätsschonenden Mechanismen wie der konstruktiven Enthaltung wurde bisher fast nie Gebrauch gemacht. Der zentrale Bereich der GASP und der GSVP bleibt somit auch weiterhin im intergouvernementalen Teufelskreis gefangen. Vorschläge zur Überwindung der Einstimmigkeit – auch unterhalb der Vertragsänderungsschwelle (z. B. via Passerelle-Klausel) – wurden auch von höchster politischer Ebene eingebracht. An ihrer Realisierbarkeit in naher Zukunft darf gezweifelt werden, zumal auch Mehrheitsentscheidungen kein Garant für Effektivität sind wie das Beispiel des mit qualifizierter Mehrheit beschlossenen Mechanismus’ zur Verteilung von Flüchtlingen belegt. Hier ist wieder auf den politischen Willen als essenzielle Grundbedingung für kollektives europäisches Handeln zu verweisen.

Ein dritter Schwächekomplex betrifft interne Probleme, die Kollateralschäden für die Handlungsfähigkeit und Glaubwürdigkeit des globalen Akteurs EU mit sich bringen. Die Debatten um die Rechtsstaatlichkeit in einigen Mitgliedstaaten werfen die Frage auf, wie überzeugend die EU rechtsstaatliche Bedingungen von Partnern einfordern kann, wenn schon die eigenen Mitglieder nicht den Clubregeln folgen und einer illiberalen Demokratie das Wort reden. Auch angesichts überfüllter Flüchtlingslager oder *Push-Backs* an europäischen Außengrenzen gerät der hohe moralische Anspruch der EU bei der weltweiten Einhaltung von Menschenrechten ins Wanken. Erschwerend kommt hinzu, dass auch die Außenpolitik der EU zunehmend politisiert und damit kontestiert wird (Costa 2019). Demonstrationen gegen TTIP oder CETA sind nur ein Beleg für die Politisierungsthese. Diese Entwicklung mag einerseits legitimatorische Vorteile mit sich bringen, hat jedoch andererseits negative Effekte auf strategische Fragen. So ist die Tatsache, dass die EU über Jahre hinweg die Länder des westlichen Balkans im europäischen „Kühlregal“ (Charles Michel) liegen ließ und damit eine strategische Blindheit gegenüber einem geopolitischen Hotspot vor ihrer Haustür an den Tag legte, nicht zuletzt der innereuropäischen Erweiterungsmüdigkeit gezollt. Das auch innenpolitischen Erwägungen geschuldete Zurückschrecken vor eigenem Engagement in der Peripherie des europäischen Kontinents (so etwa in Syrien oder Libyen) führt zu der wenig erbaulichen Situation, dass diese Regionen inzwischen von rivalisierenden Großmächten mit eigenen ordnungspolitischen Vorstellungen dominiert werden (Kaim und Kempin 2020).

### Chancen

Nach der Bestandsaufnahme der akteursspezifischen Stärken und Schwächen fragt die SWOT-Analyse nach den Chancen und Risiken, die sich im Wettbewerbsumfeld bieten. Will man aus dem aktuellen globalen Umfeld eine Chance für die EU-Außenpolitik ableiten, so ist es wohl die, dass die gegenwärtige Verfasstheit des internationalen Systems die EU-Mitgliedstaaten gewissermaßen zu außenpolitischer Kooperation zwingt. Allein verfügt kein EU-Mitgliedstaat über Gestaltungmacht im Wettbewerb der Großmächte. Ein bekanntes kenianisches Sprichwort besagt nicht umsonst: „Wenn Elefanten kämpfen, leidet das Gras“. Der Brexit zeigt die Grenzen der nationalen Souveränität auf und lässt den Slogan ‚Take back control‘ zur Chimäre werden. Das Ausscheiden des Vereinigten Königreichs aus der EU zog keinen Dominoeffekt nach sich, sondern hatte eine abschreckende Wirkung auf andere potenzielle Aussteiger und beförderte die vom französischen Staatspräsidenten angestoßene Debatte zur europäischen Souveränität als politisches Programm zur Rettung europäischer Gestaltungsmacht. Das internationale Umfeld bietet den europäischen Staaten die Chance, souverän Entscheidungen zu treffen und zu handeln – wenn sie es gemeinsam tun und die EU als Souveränitätsverstärker nutzen.

Eine zweite wichtige Chance ergibt sich daraus, dass der globale Wettbewerb der Systeme durchaus eine Nische für das Geschäftsmodell der EU bietet: Die Welt der G2 mit den stark marktfokussierten USA und der staatsfokussierten Volksrepublik China eröffnet Leerstellen, die die EU füllen kann. Als ein Beispiel sei auf den Umgang mit Daten und künstlicher Intelligenz verwiesen, der in der anstehenden digitalen Dekade entscheidend sein wird. Die EU kann mit ihrem Vorhaben eines „human-centred digital ecosystem“ (Margrethe Vestager) mit *small data* und Datenschutzbestimmungen, die schon jetzt einen weltweiten Standard darstellen, in der globalen digitalen Transformation einen dritten Weg bieten zwischen den USA und China und damit den spezifisch europäischen Zugang als Wettbewerbsvorteil ausbauen.

### Risiken

Das globale System in seiner aktuellen Verfasstheit kann die EU, die eine „structural foreign policy“ (Keukeleire und Delreux 2014, S. 28 f.) mit strukturprägender Wirkung im Sinne der oben angesprochenen Innen-Außen-Analogie anstrebt, nicht zufriedenstellen. Der noch zu Beginn des Jahrtausends erhoffte weltweite Siegeszug der liberalen Demokratie ist nicht eingetreten. Der Aufstieg autoritärer Mächte, vor allem Chinas, impliziert, dass eine demokratische Grundstruktur keine notwendige Voraussetzung für wirtschaftlichen Erfolg ist. Die EU sieht sich verstärkt mit „strongmen“ (Kribbe 2020) und deren autoritären Ordnungsvorstellungen konfrontiert. Besonders in der unmittelbaren Peripherie, wo die EU in vielen Bereichen auf Kooperation mit ihnen angewiesen ist, stellen diese „strongmen“ die Union vor große Herausforderungen, so etwa in der Türkei, in Belarus oder in Russland. Geographisch stellt die südliche und östliche Nachbarschaft der EU, die sich – anders als noch 2004 avisiert – nicht zum „ring of friends“ (Romano Prodi), sondern zum „ring of fire“ (The Economist 2014) entwickelte, zweifelsohne das größte Risiko dar.

Weitere gravierende Risiken resultieren aus der Fülle an Herausforderungen, den ‚problems without passport‘, denen sich die EU-Außenpolitik gegenübersieht: Terrorismus, die Verbreitung von Massenvernichtungswaffen, Cyber-Kriminalität, Pandemien, organisierte Kriminalität, Menschenhandel, illegale Migration und hybride Bedrohungen zählen ebenso dazu wie Umweltzerstörungen oder die planetare Bedrohung des Klimawandels. Das aus der Pandemie bekannte Präventionsparadox („There is no glory in prevention.“) findet sich auch im Umgang mit dem Klimawandel und ist für Demokratien, welche auf Unterstützung und Wiederwahl angelegt sind, besonders relevant. Erschwerend kommt hinzu, dass die Klimawandelfolgen maßgeblich in der Zukunft liegen, der klimapolitische „leadiator“ EU (Bäckstrand und Elgström 2013) die Weichen für die Anpassungsmaßnahmen zusammen mit anderen Akteuren jedoch jetzt stellen muss.

## 2001 bis 2021: Die EU zwischen Schlummertaste und Geopolitik

Insgesamt lässt die SWOT-Kurzanalyse Licht und Schatten der EU-Außenpolitik erkennen. Was überwiegt? Betrachtet man zur Beantwortung dieser Frage die Genese des globalen Akteurs von 2001 bis 2021, so ist ein deutlicher Aufwärtstrend zu verzeichnen, was die Weltpolitik- und damit Zukunftsfähigkeit der EU angeht. Als die Flugzeuge in den USA am 11. September 2001 ihre Ziele trafen, umfasste die EU fünfzehn Mitgliedstaaten und bereitete sich auf die Einführung des Euro und die *Big Bang*-Erweiterung um zehn neue Mitgliedstaaten vor. Die ESVP hatte gerade zweiten Geburtstag gefeiert und war noch nicht operativ einsetzbar. Der Vertrag von Nizza war schon unterzeichnet, die EU funktionierte aber noch auf der Basis des Vertrags von Amsterdam, in welchem unter anderem das Amt des Hohen Vertreters für die GASP eingeführt worden war, der sich seine Zuständigkeit damals noch mit der rotierenden Ratspräsidentschaft und dem Kommissar für Außenbeziehungen teilen musste. Es folgte nach den langen Geburtswehen des Post-Nizza-Prozesses 2009 der Vertrag von Lissabon, der die EU-Außenpolitik zwar nicht revolutionierte, aber doch neu sortierte und zunehmend brüsselisierte. Das Lissabonner Upgrade fiel mit einer Stagnationsphase der EU-Außenpolitik zusammen, in der Staatsschulden, Rettungsschirme und Fiskalpakt außenpolitische Themen überlagerten, so dass der Vertrag sein Potenzial nicht entfalten konnte. Als der Arabische Frühling sein demokratisches Band durch die Lüfte flattern ließ, verharrte die EU angesichts der Instabilitäten vor ihrer Haustür in einer Schockstarre. Die *Battlegroups* kamen nicht zum Einsatz. Die europäische Antwort auf die ‚Arabellion‘ war unkoordiniert. Obwohl die Alarmglocken der Unsicherheit und Instabilität in ihrer unmittelbaren Nachbarschaft läuteten, drückten die EU-Mitgliedstaaten die Schlummertaste und drehten sich unter der warmen Decke des US-amerikanischen *Security Provider *zum Weiterschlafen um. Im weiteren Verlauf erhöhte sich die Schlagzahl der außenpolitischen Weckrufe. Die Annexion der Krim 2014 kann als geopolitischer Erweckungsmoment für die EU betrachtet werden. Vollends wachgerüttelt wurden die EU-Mitgliedstaaten sodann im Jahr 2016 mit der Wahl Donald Trumps zum US-Präsidenten. Er bezeichnete die EU nicht nur offen als „foe“, als „as bad as China, just smaller“, sondern griff mit dem Rückzug der USA aus dem Pariser Klimaabkommen und dem *Joint and Comprehensive Plan of Action* mit dem Iran auch die Inkarnation jüngerer außenpolitischer Erfolge der EU frontal an. Der Brexit wirkte als weiterer Katalysator und beschleunigte gerade im GSVP-Bereich neue Initiativen (so etwa PESCO, CARD, EDF, Europäische Friedensfazilität). Die selbst ernannte „geopolitische Kommission“ unter Ursula von der Leyen verfolgt seit 2019 einen hohen Gestaltungsanspruch, der alle Dimensionen des außenpolitischen Mosaiks umfasst. Aufbauend auf der *EU Global Strategy* von 2016 ist in den letzten Jahren eine deutliche Strategisierung der EU-Außenpolitik zu konstatieren, erkennbar zum Beispiel in der neuen Formel gegenüber China, das für die EU gleichzeitig Partner, Wettbewerber und strategischen Rivalen darstellt. Die Abhängigkeitserfahrungen von China während der COVID-19-Pandemie haben der strategischeren Ausrichtung und den Rufen nach europäischer Souveränität und strategischer Autonomie weiteren Schub gegeben.

Aufgrund der gebotenen Kürze kann der Beitrag nur tentative Antworten auf die Frage nach der Zukunftsfähigkeit der EU geben. Extrapoliert man die hier in groben Zügen skizzierte Entwicklung der letzten 20 Jahre in die Zukunft, ist anzunehmen, dass die EU die zahlreichen Weckrufe nicht ungehört verschallen lassen und stattdessen aus dem Tiefschlaf der außen- und sicherheitspolitischen Tatenlosigkeit, Uneinigkeit und Ambitionslosigkeit endgültig erwachen könnte. „It’s time to wake up and smell the post-American coffee“, folgert Shapiro (2021) als Lehre aus Afghanistan. Mit Mali droht der EU ein afrikanisches Afghanistan. Dass die USA an dieser Region kein vitales Interesse haben, haben sie längst klar gemacht. Mit dem neuen trilateralen Bündnis AUKUS, das die USA überraschend mit Australien und dem Vereinigten Königreich eingegangen sind, hat der Wecker erneut geklingelt, der die EU daran erinnert, dass sie selbst ihre Rolle im asiatischen Jahrhundert noch finden muss. Klar ist, dass entscheidende sicherheitspolitische Weichenstellungen jetzt anstehen, sei es beim strategischen Kompass, der Teambuilding-Maßnahme in Sachen gemeinsamer strategischer Kultur, beim Verteidigungsgipfel unter französischer Ratspräsidentschaft 2022, wo unter anderem die ventilierte schnelle Eingreiftruppe auf dem Tapet liegt, oder auch im Rahmen der Konferenz zur Zukunft der EU, bei welcher die globale Rolle der EU eine der zentralen Fragen darstellt. Die EU-Mitgliedstaaten werden nicht umhinkommen, in den nächsten Monaten die außenpolitischen Gretchenfragen z. B. nach den prioritären Handlungsarenen der EU-Außenpolitik zu beantworten, vor denen sie sich lange gedrückt haben.

In jedem Fall wird die EU auch weiterhin eine „strange superpower“ (The Economist 2020) bleiben – in anderen Worten: eine kollektive Weltmacht sui generis, deren Gewicht und Gestaltungsmacht in den unterschiedlichen Dimensionen des außenpolitischen Mosaiks differiert. Wenn sie ernstzunehmende Spielerin und nicht Spielfeld oder gar Spielball der Geopolitik im 21. Jahrhundert sein möchte, kann sie sich „strategisches Schlafwandeln“ (Josep Borrell) nicht mehr leisten, sondern muss den eingeschlagenen Weg fortführen und das gesamte Potenzial ihres außenpolitischen Mosaiks strategisch und zukunftsfähig zur Geltung bringen.

## Footnotes

1 Vgl. Abschn. 4.
